# Effect of aluminum accumulation on bone and cardiovascular risk in the current era

**DOI:** 10.1371/journal.pone.0284123

**Published:** 2023-04-20

**Authors:** Cinthia E. M. Carbonara, Noemi A. V. Roza, Kelcia R. S. Quadros, Renata A. França, André B. A. Esteves, Celia R. Pavan, Joaquim Barreto, Luciane M. dos Reis, Vanda Jorgetti, Andrei C. Sposito, Rodrigo Bueno Oliveira

**Affiliations:** 1 Nephrology Division, School of Medical Sciences, University of Campinas (Unicamp), Campinas, Brazil; 2 Laboratory for Evaluation of Mineral and Bone Metabolism in Nephrology (LEMON), School of Medical Sciences, University of Campinas (Unicamp), Campinas, Brazil; 3 Laboratory of Atherosclerosis and Vascular Biology, Cardiology Division, School of Medical Sciences, University of Campinas (Unicamp), Campinas, Brazil; 4 Laboratory of Renal Pathophysiology, Department of Internal Medicine, School of Medicine, University of São Paulo, São Paulo, Brazil; Medical College of Wisconsin, UNITED STATES

## Abstract

**Background:**

The prevalence of aluminum (Al) intoxication has declined over the past 3 decades. However, different groups still report on the diagnosis of Al in bone. Prolonged and low-intensity exposures to Al may not be captured by serum Al measurements, preventing its proper diagnosis. We hypothesize that bone Al accumulation may be related to bone and cardiovascular events in the current Era.

**Aims:**

To detect the diagnosis of bone Al accumulation; to explore bone and cardiovascular consequences of Al accumulation.

**Methods:**

This is a sub-analysis of *The Brazilian Registry of Bone Biopsy*, a prospective, multicentre cohort, with a mean follow-up of 3.4 years, including patients with CKD undergoing bone biopsy; bone fracture and major cardiovascular events (MACE) were adjudicated; Al accumulation was identified by solochrome-azurine staining; history of previous Al accumulation was registered based on information provided by the nephrologist who performed the bone biopsy; bone histomorphometry parameters, clinical data, and general biochemistry were registered.

**Results:**

275 individuals were considered; 96 (35%) patients have diagnosed with bone Al accumulation and were younger [50 (41–56) *vs*. 55 (43–61) years; p = 0.026], had lower body mass index [23.5 (21.6–25.5) *vs*. 24.3 (22.1–27.8) kg/m^2^; p = 0.017], higher dialysis vintage [108 (48–183) *vs*. 71 (28–132) months; p = 0.002], presented pruritus [23 (24%) *vs*. 20 (11%); p = 0.005], tendon rupture [7 (7%) *vs*. 3 (2%); p = 0.03) and bone pain [2 (0–3) *vs*. 0 (0–3) units; p = 0.02]. Logistic regression reveals that prior bone Al accumulation [OR: 4.517 (CI: 1.176–17.353); p = 0.03] and dialysis vintage [OR: 1.003 (CI: 1.000–1.007); p = 0.046] as independent determinants of bone Al accumulation; minor perturbations in dynamic bone parameters and no differences in bone fractures rate were noted; MACE was more prevalent in patients with bone Al accumulation [21 (34%) *vs*. 23 (18%) events; p = 0.016]. Cox regression shows the actual/prior diagnosis of bone Al accumulation and diabetes *mellitus* as independent predictors for MACE: [HR = 3.129 (CI: 1.439–6.804; p = 0.004) and HR = 2.785 (CI: 1.120–6.928; p = 0.028].

**Conclusions:**

An elevated proportion of patients have bone Al accumulation, associated with a greater prevalence of bone pain, tendon rupture, and pruritus; bone Al accumulation was associated with minor perturbations in renal osteodystrophy; actual/prior diagnosis of bone Al accumulation and diabetes *mellitus* were independent predictors for MACE.

## Introduction

The era of aluminum (Al) intoxication as the cause of dialysis dementia epidemic ended in the 80s-90s of the last century, with the incorporation of new technologies for treating water used for hemodialysis and the proscription of aluminum-based drugs [1–4].

The understanding of the causes involved in Al contamination and the use of desferrioxamine, this complication decreased in medical practice, limited to some anecdotal case report descriptions or in accidental conditions [5–7]. However, different groups from China, Uruguay and Argentina still report on case series and cohorts with the diagnosis of bone Al accumulation and complications related to Al load [8–10].

In Brazil, despite the adoption of international guidelines to prevent Al intoxication [11], we also still detect Al in bone [12–14]. Here, due to a unique characteristic, bone biopsies depend on the medical indication and the patient’s acceptance, since bone biopsy is analyzed in reference centers at no cost to the patient. This practice allows identifying cases of bone Al accumulation over the decades by the gold standard method: bone biopsy stained by solochrome azurine [4, 15].

The incongruence of continued bone Al detection despite reduced Al exposure deserves consideration of two main possibilities: (1) prolonged and low-intensity exposures to Al sources may not be adequately captured by measurements of serum Al levels, since it does not reflect deposition in tissue, preventing its proper diagnosis; [4, 15] (2) the high incidence and prevalence of chronic kidney disease (CKD) cause a global burden for healthcare systems, with an elevated disparity in treatment access and heterogeneous standards of quality in provided care [16–22].

These facts lead us to hypothesize that bone Al accumulation still occurs in a considerable proportion of the population with CKD, not as a classical presentation syndrome with systemic signs of intoxication as it was in the past; instead, with chronic effects in bone, mineral metabolism, and may be in other organs, such as the heart.

Of note, preclinical evidence suggests that Al has cardiac toxicity through alterations in oxidative stress [23, 24], apoptosis [23], lipoproteins [25], myocardial inflammatory cytokines, and vascular reactivity [26, 27], it seems to be reasonable to speculate that Al accumulation in patients with CKD can affect the cardiovascular system. There is a lack of data regarding Al accumulation and its consequences in the cardiovascular system of patients with CKD.

This study aims are to detect the actual frequency of diagnosis of bone Al accumulation and to explore bone and cardiovascular consequences of bone Al accumulation in a prospective cohort of Brazilian patients undergoing bone biopsy, through a pre-specified analysis of *The Brazilian Registry of Bone Biopsy* (REBRABO) [28].

## Material and methods

### Study design and patient selection

This study was conducted as a sub-analysis of REBRABO [28], a prospective, national multicentre cohort, which consisted of the longitudinal follow-up of patients with chronic kidney disease according to the Kidney Disease Improving Global Outcomes (KDIGO) [29], all adults, undergoing bone biopsy. The main objective of this analysis was to detect the diagnosis of bone Al accumulation and to describe clinically relevant associations in the context of renal osteodystrophy. A secondary objective was to explore bone and cardiovascular consequences of bone Al accumulation.

During the period from August 2015 to December 2021, 511 patients underwent bone biopsy and had their data included in REBRABO. Patients who lost their follow-up (N = 111), or have not bone biopsy report (N = 40), or estimated glomerular filtration rate > 90 mL/min (N = 28), or have not signed their consent (N = 24), or have bone fragment inadequate for diagnostic (N = 23), or bone biopsy indicated by another specialty than Nephrology (N = 6), or < 18 years old (N = 4) were excluded from this analysis.

The baseline was defined as the time when the patient underwent bone biopsy. The prospective analysis included data from patients who completed at least 12 months of follow-up. Over a mean follow-up of 3.4 years (693 to 1508 days), the following events were adjudicated: bone fracture and major cardiovascular events (unstable angina, nonfatal acute myocardial infarction, elective or emergency coronary revascularization, transient ischemic attack, stroke, and cardiovascular death).

Written informed consent was obtained from all subjects and patients; the local ethics committee approved the study protocol under numbers CAAE 4131141.6.0000.5404, and the clinical and research activities being reported are consistent with the Declaration of Helsinki.

### Clinical, demographic, and laboratory data

The clinical, demographic, and laboratory data were collected at baseline and follow-up using standard electronic forms available at the REBRABO web system. The baseline data were entered by a nephrologist who performed the bone biopsy and validated by a single researcher. Data about previous Al accumulation, as well, as those related to clinical symptoms (e.g., bone pain, pruritus, myalgia) were based on information from the nephrologist who performed the bone biopsy. Symptoms and complications related to renal osteodystrophy were scored as the following: bone pain intensity (pain visual analog scale; intensity from 0 to 5); for pruritus, myalgia, tendon rupture, bone deformity, and bone fractures, the information was categorized into “yes” or “no”; The follow-up data were obtained and validated by researchers. These data were collected by telephone call with the nephrologists, the dialysis unit’s staff, and the patients.

In this sub-analysis of REBRABO, the following data were considered: clinical—age, gender, ethnicity, diagnosis of diabetes *mellitus*, CKD etiology, residual estimated glomerular filtration rate, dialysis vintage, and modality, prior cardiovascular disease (coronary disease, myocardial infarct, or stroke), prior parathyroidectomy, prior diagnosis of bone Al accumulation, symptoms and complications related to renal osteodystrophy (bone pain intensity, pruritus, myalgia, tendon rupture, bone deformity, and bone fractures); laboratory—serum levels of total calcium, phosphate, parathormone, alkaline phosphatase, 25-hydroxyvitamin D and hemoglobin.

### Bone biopsy and tissue analysis

Bone biopsy was indicated by medical reasons (main indications: persistent bone pain, unexplained hypercalcemia or hyperphosphatemia, non-traumatic bone fractures, and suspicion of Al accumulation) or research protocol. Bone fragments were obtained via transiliac bone biopsies using an electrical trephine after pre-labeling with tetracycline (3 days) administered over two separated periods 10 days apart. Undecalcified bone fragments were submitted to standard processing for histological studies [30].

Al bone content was identified by solochrome azurine staining. We examined all fields of the trabecular bone samples under the magnification of x125. We considered the diagnosis of bone Al accumulation when 30% or more of the surface of the trabecular bone was covered by Al. This relatively high cutoff allows for increasing the specificity, avoiding false-positive diagnosis.

Bone histomorphometry was conducted using the Osteomeasure software (Osteometrics Inc., Atlanta, Ga., USA). Static and dynamic parameters were analyzed following the Standards of the American Society of Bone and Mineral Research [31]. Bone sections were stained with toluidine blue. The references range used for static and dynamic parameters was obtained from dos Reis LM *et al*. and Vedi S *et al*., respectively [32, 33]. The samples from individual patients were classified as having osteitis fibrosa, mixed uremic osteodystrophy, adynamic bone disease, osteomalacia, and osteoporosis [34].

### Statistical analysis

The continuous variables are reported as the means ± SD or medians and interquartile intervals. The categorical data are reported as frequencies and percentages. Mann-Whitney test, X^2^, Fisher exact test, and Z-test were applied to compare patients with and without bone Al accumulation, as appropriate. Binary logistic regression was performed to identify the independent determinants of the diagnosis of bone Al accumulation. Covariates and factors selected from the univariate regression were body mass index, residual renal function (GFR < 15 mL/min, reference), dialysis modality (reference, peritoneal dialysis), dialysis vintage, calcium salts use, and prior bone Al accumulation. Cox regression analysis, enter method, was undertaken with MACE-dependent variables. The following independent covariates and factors were selected from univariate regression analysis: diagnostic of diabetes *mellitus*, actual/prior diagnosis of bone Al accumulation, and previous history of cardiovascular disease. A Kaplan-Meier curve with a log-rank test was used to represent survival related to MACE. Statistical analyses were performed using SPSS 22.0 (SPSS Inc., Chicago, IL). A two-sided p-value < 0.05 was considered statistically significant.

## Results

Data from 275 individuals were considered in our analysis. Patients were relatively young and all of them had CKD: 221 (80%) were on hemodialysis, 27 (10%) on peritoneal dialysis, and 27 (10%) on conservative management. Detailed clinical and biochemical baseline characteristics are summarized in Table 1.

**Table 1:** General clinical and biochemical data according the diagnosis of bone aluminum (Al) accumulation.

	All	No Al accumulation	Al accumulation	p
	(N = 275)	(N = 179)	(N = 96)	
Age (years)	52 (42–60)	55 (43–61)	50 (41–56)	**0.026**
Body mass index (kg/m^2^)	24.1 (21.9–27.4)	24.3 (22.1–27.8)	23.5 (21.6–25.5)	**0.017**
Gender (male; N, %)	143 (52)	96 (54)	47 (49)	0.46
Ethnicity (Caucasian; N, %)	118 (43)	83 (46)	35 (37)	0.11
Diabetes mellitus (N, %)	39 (14)	27 (15)	12 (12)	0.55
Prior cardiovascular disease (N, %)	27 (10)	16 (9)	11 (11)	0.50
CKD etiology				0.14
Hypertension (N, %)	78 (28)	56 (31)	22 (23)	
Chronic GN (N, %)	65 (24)	37 (21)	28 (29)	
Diabetes *mellitus* (N, %)	37 (13)	25 (14)	12 (12)	
Residual RF (eGFR < 15 mL/min; N, %)	244 (89)	149 (83)	95 (99)	**0.0001**
Dialysis vintage (months)	84 (36–146)	71 (28–132)	108 (48–183)	**0.002**
Dialysis modality (hemodialysis; N, %)	221 (80)	130 (73)	91 (95)	**0.02**
Hemoglobin (g/dL)	11.5 (10.3–13)	11.5 (10.4–13.1)	11.4 (10.2–12.8)	0.37
Total calcium (mg/dL)	9.3 (8.6–9.8)	9.2 (8.6–9.8)	9.3 (8.6–9.9)	0.66
Phosphate (mg/dL)	5 (3.9–6.5)	4.6 (3.8–6)	5.6 (4.5–7.1)	**0.0001**
Parathormone (pg/mL)	234 (65–733)	253 (83–780)	173 (37–656)	**0.04**
Alkaline phosphatase (IU/L)	120 (79–217)	117 (81–210)	129 (71–220)	0.62
25-vitamin D (ng/mL)	29.6 (20.5–38)	30.7 (22–38)	28.3 (19.3–40)	0.87

GN, glomerulonephritis; RF, residual function. Continuous variables are expressed as median and interquartile range (25th–75th).

Ninety-six patients were diagnosed with bone Al accumulation. Serum Al levels were 10.6 (5.5–18.4) μg/L, and were not different between those with or without bone Al accumulation (p = 0.55); 10 (17%) patients had serum Al levels > 30 μg/L. Compared with patients without a diagnosis of Al accumulation, patients with the diagnosis of Al intoxication were younger [50 (41–56) *vs*. 55 (43–61) years; p = 0.026] and had a lower body mass index [23.5 (21.6–25.5) *vs*. 24.3 (22.1–27.8) kg/m^2^; p = 0.017]. They had higher dialysis vintage [108 (48–183) *vs*. 71 (28–132) months; p = 0.002] and serum phosphate [5.6 (4.5–7.1) *vs*. 4.6 (3.8–6) mg/dL; p = 0.0001], and lower parathormone [173 (37–656) *vs*. 253 (83–780) pg/mL; p = 0.04] levels. Almost all patients with bone Al accumulation were on hemodialysis treatment [91 (95%)]. Of note, we observed a higher proportion of calcium salts use among patients with bone Al accumulation [33 (34%) vs. 37 (21%), p = 0.01]. The use of Al-based P binders was not observed.

At baseline patients with bone Al accumulation presented more clinical symptoms and complications related to renal osteodystrophy than those without. They presented a higher frequency of pruritus [23 (24%) *vs*. 20 (11%); p = 0.005], episodes of tendon rupture [7 (7%) *vs*. 3 (2%); p = 0.03), and more intensity of bone pain [2 (0–3) *vs*. 0 (0–3) units; p = 0.02]. They had more history of prior parathyroidectomy [26 (27%) *vs*. 20 (11%); p = 0.001] and prior diagnosis of bone Al accumulation [12 (12%) *vs*. 3 (2%); p = 0.0001]. There were no differences in the frequency of bone deformities [20 (21%) *vs*. 22 (12%); p = 0.06], myalgia [32 (33%) *vs*. 44 (25%); p = 0.12], and bone fractures [19 (20%) *vs*. 28 (16%); p = 0.38].

Logistic regression reveals the diagnosis of prior bone Al accumulation [OR: 4.517 (CI: 1.176–17.353); p = 0.03] and dialysis vintage [OR: 1.003 (CI: 1.000–1.007); p = 0.046] as independent determinants of actual diagnosis of bone Al accumulation.

One hundred and ten patients had bone histomorphometric analysis. Normal bone histology was detected in 4 patients, one of them in a patient with the diagnosis of bone Al accumulation. The same proportion of renal osteodystrophy types was observed comparing patients with and without a diagnosis of bone Al accumulation (p = 0.11): fibrous osteitis [7 (29%) *vs*. 28 (33%)], mixed uremic osteodystrophy [6 (25%) *vs*. 9 (10%)], adynamic bone disease [9 (37%) *vs*. 46 (53%), osteomalacia [1 (4%) *vs*. 0(0%)]; and osteoporosis [14 (58%) vs. 49 (57%), p = 0.905].

Histomorphometric bone parameters according to the diagnosis of bone Al accumulation show no significant differences in structural, resorption, and formation parameters. In dynamic parameters, only mineralization lag time was higher in patients with the diagnosis of bone Al accumulation (Table 2).

**Table 2:** Histomorphometric bone parameters according to the diagnosis of bone aluminum (Al) accumulation.

	All	No Al accumulation	Al accumulation	p
	(N = 110)	(N = 86)	(N = 24)	
BV/TV (%)	20.2 (20.1–25)	20.4 (16.1–28)	20.2 (15.6–26)	0.50
Tb.Th (μm)	123 (110–140)	124 (112–144)	120 (103–135)	0.19
Tb.Sp (μm)	464 (364–594)	466 (364–603)	460 (369–581)	0.82
Tb.N (mm/mm)	1.64 (1.38–1.97)	1.69 (1.4–1.9)	1.61 (1.31–1.97)	0.75
OV/BV (%)	1.52 (0.62–4.06)	1.51 (0,61–3,58)	1.61 (0.8–5.33)	0.40
O.Th (μm)	7.1 (5.4–8.7)	7 (5.2–8.6)	7.5 (5.7–8.8)	0.53
OS/BS (%)	15.1 (7.5–30.5)	15.1 (7.5–29.6)	18.1 (8.6–34)	0.42
Ob.S/BS (%)	2.77 (1.36–7.22)	2.39 (1.33–6.47)	5.8 (1.65–8.03)	0.12
ES/BS (%)	3.8 (2.44–7.24)	3.77 (2.28–6.43)	5.40 (2.51–7.57)	0.37
Oc.S/BS (%)	0.27 (0.09–0.62)	0.27 (0.09–0.63)	0.25 (0.08–0.57)	0.77
MS/BS (%)	5.73 (2.71–10.14)	5.1 (2.52–10.56)	6.87 (4.08–9.37)	0.83
MAR (μm/dia)	0.68 (0.48–0.94)	0.65 (0.48–0.94)	0.72 (0.61–1.08)	0.35
BFR/BS (μm^3^/μ^2^/d)	0.030 (0.011–0.082)	0.030 (0.014–0.090)	0.034 (0.000–0.073)	0.31
Aj.Ar (μm/d)	0.305 (0.170–0.577)	0.319 (0.189–0.617)	0.282 (0.116–0.425)	0.19
Mlt (d)	32.58 (17.28–68.08)	25.06 (13.91–58.81)	60.5 (21.6–285)	**0.012**
Fb.V/TV (%)	0.039 (0.009–0.171)	0.043 (0.013–0.277)	0.014 (0.000–0.080)	**0.018**

BV/TV, bone volume/tissue volume; Tb.Th, trabecular thickness; Tb.Sp, trabecular separation; trabecular number; OV/BV, osteoid volume/bone volume; O.Th, osteoid thickness; OS/BS, osteoid surface/bone surface; Ob.S/BS, osteoblasts surface/bone surface; ES/BS, eroded surface/bone surface; Oc.S/BS, osteoclast surface/bone surface; MS/BS, mineralized surface/bone surface; MAR, mineral apposition rate; BFR/BS, bone formation rate/bone surface; Aj.Ar, adjusted area; Mlt, mineralization lag time; Fb.V/TV, fibrosis volume/tissue volume.

### Exploratory analysis of bone and cardiovascular consequences related to bone Al accumulation

Over a mean follow-up of 3.4 years (693 to 1508 days), bone fracture and MACE were adjudicated. The same proportion of bone fractures was registered between those with or without bone Al accumulation [8 (13%) *vs*. 20 (16%); p = 0.63].

MACE in patients with bone Al accumulation reached 21 (34%) events compared with 23 (18%) in those patients without (p = 0.016). Patients who experienced MACE had lower serum levels of hemoglobin (11.3 ± 2.1 *vs*. 12 ± 2.1; p = 0.026), more diagnosis of diabetes *mellitus* [11 (25%) *vs*. 15 (10%); p = 0.013], history of cardiovascular disease [8 (18%) vs. 8 (5%); p = 0.008], and actual/prior diagnosis of bone Al accumulation [22 (50%) vs. 42 (29%); p = 0.01].

**Fig 1:**
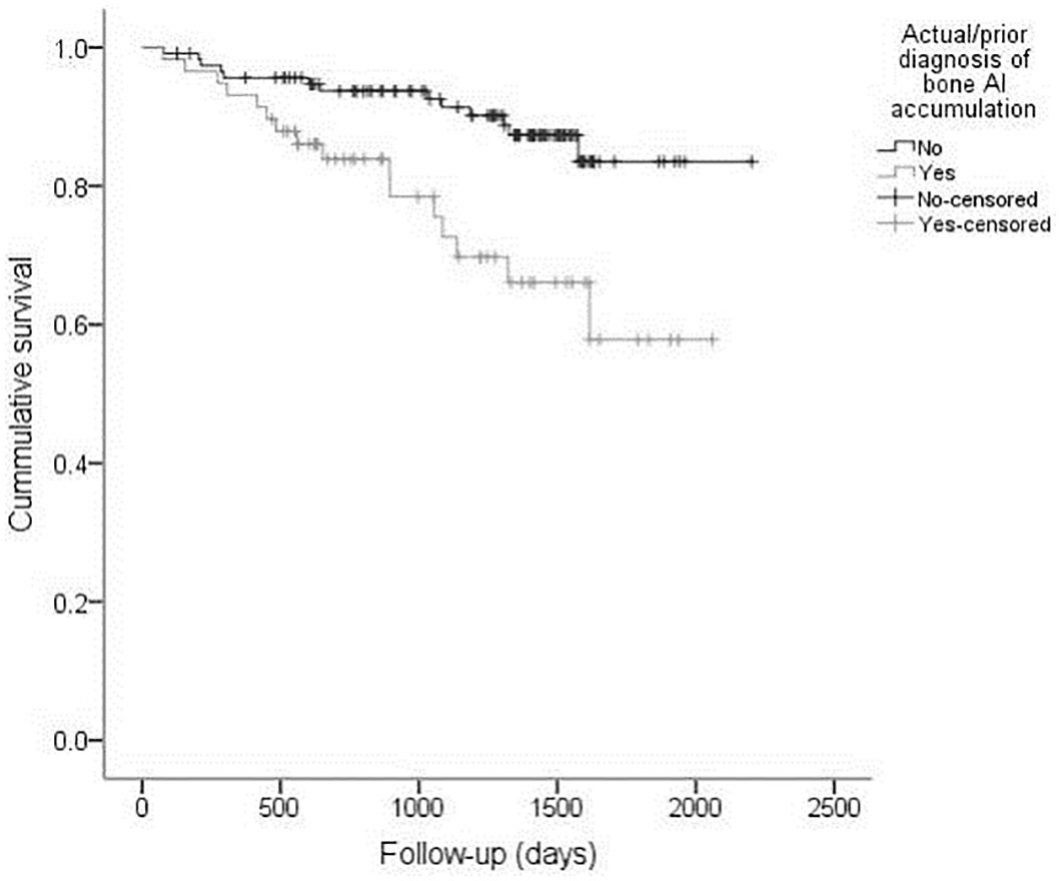
Kaplan-Meier curve for MACE according to actual/prior diagnosis of bone Al accumulation (log-rank; p = 0.002).

Cox regression analysis shows the actual/prior diagnosis of bone Al accumulation and diabetes *mellitus* as independent predictors for MACE: 3.129 (CI: 1.439–6.804; p = 0.004) and HR = 2.785 (CI: 1.120–6.928; p = 0.028] (Fig 1).

## Discussion

Our study shows the following main findings: (1) an elevated proportion of patients have the diagnosis of bone Al accumulation, which was associated with a greater prevalence of bone pain, tendon rupture, and pruritus; (2) bone Al accumulation was associated with minor perturbations in bone dynamic parameters; none difference in static bone parameters, the proportion of classical renal osteodystrophy types and bone fractures were observed; (3) actual/prior diagnosis of bone Al accumulation and diabetes *mellitus* were independent predictors for MACE.

After the 90s of the last century, severe symptoms reflecting Al intoxication disappeared because adoption of international guidelines to avoid contamination [2–4, 11]. The diagnosis has become almost exclusively through the serum Al measurements and epidemiological data from American studies concludes that Al intoxication is an uncommon finding. Two retrospective cohorts analyzed more than seven thousand serum Al measurements in patients on hemodialysis and peritoneal dialysis from 2000 to 2009, with 2.1% of the samples altered and without significant clinical manifestations [35, 36].

Measurement of serum Al does not reflect the tissue load accurately [15, 37]. Van Landeghem and collaborators showed that patients with iron overload and normal levels of serum Al can have bone disease related to this metal [15]. Serum measurements can be useful to reveal acute exposures to this metal, but its effectiveness in other scenarios is questionable.

This limitation in actual diagnostic practice in conjunction with prolonged and low-intensity exposures to Al sources might explain the elevated proportion of patients presenting the diagnosis of bone Al accumulation in our cohort and from others studies [8–10, 12–14]. Since heterogeneous standards of water quality for hemodialysis treatment around the globe is not negligible it is plausible to expect these findings in other populations.

There are several sources of Al, besides the treaded water for dialysis. Medications used in patients undergoing dialysis may contain Al, especially in intravenous form, such as dipyrone, erythropoietin, and iron preparations, as well calcium salts [38]. The impact of this contamination is unknown. Besides these drugs, calcium citrate and ferric citrate hydrate can increase Al absorption from the diet. Of note, in our cohort, we did not detect any patient in the use of calcium citrate or ferric citrate hydrate. As for diet, data on intestinal absorption of Al in healthy subjects reveal absorption of small quantities (0.06–0.1%). Factors that may influence absorption and its bioavailability are compounds that bind to Al in the intestinal lumen, gastric acidity, and the hardness of water consumed [39].

In our cohort, Al in bone was associated with bone pain, tendon rupture, and pruritus. In a study with 866 patients under hemodialysis, serum Al levels were identified as an independent predictor of pruritus. The authors claim the allergenic nature of Al that can induce immune reactions [8]. Severe symptoms of Al intoxication were not declared in our cohort, as were noted in the past.

Likewise, Al in bone was not associated with osteomalacia and adynamic bone disease, as were reported in the 90s from the last century. Al accumulation was equally distributed among the classical types of renal osteodystrophy and just minor perturbation in mineralization parameters was detected. The others histomorphometric parameters did not differ in the presence of the metal deposits in the bone. Although previous scientific evidence suggest that low bone turnover provides a possible physiological link between vascular calcification and MACE, evidence on this subject is not conclusive due to several factors such as bias in studies sample selection and size, the incidence of bone fractures, and other important hormonal changes like as in FGF23 levels [40–43].

We detected 28 bone fractures along 3.4 years of follow-up (29.8 bone fractures/1000 patients/years). This incidence is according to report by the literature for CKD patients stage 5 and 5 on dialysis (17.2 to 46.3/1000 patients/year in women and 10.6 to 24.3/1000 patients/year in men) [44]. Of note, bone Al accumulation in our sample was not associated with the incidence of bone fractures. The observed increase in Mlt in patients with bone Al accumulation could traduce mild disturbance on bone mineralization without significantly affecting bone volume, a surrogate marker of bone fractures in CKD patients.

In this study, we observed that bone Al accumulation was independently associated with major adverse cardiovascular events. In the past, the association between serum Al levels and mortality in CKD patients was documented [45, 46]. Tzu-Lin *et al*. reported the independent relation between the proportion of the heart size to the thoracic diameter and serum Al levels in patients on hemodialysis [47].

The advancement of molecular research has demonstrated myocardial and vascular toxicity by Al, as this metal can mediate oxidative damage, affects vascular reactivity, and the apoptosis of cardiomyocytes [23–27]. Zhou *et al*. administered different doses of AlCl_3_ solution by intraperitoneal injection and measured the apoptosis of cardiomyocytes and the expression of apoptosis-related proteins. They observed that TUNEL staining showed more apoptosis and less expression of Bcl-2 in animals with Al exposure compared with those without Al exposure [23]. Another hypothesis is that Al deposition can stimulate the chronic inflammatory state and provide atherosclerosis [48].

This study has limitations. It is essentially a description of a longitudinal cohort and is not a random analysis. The extrapolation of these findings to the general dialysis community is uncertain. Bone biopsy was indicated exclusively by assistant Nephrologists, who also, entered clinical information. Prior knowledge of aluminum intoxication by the Nephrologists who indicated and performed the bone biopsy, as well, as their understanding of provided clinical information constitutes an unavoidable bias. Laboratory tests were not centralized in a single laboratory with research-controlled quality parameters; a limited number of patients had serum Al levels available (N = 57); bone fractures and MACE were adjudicated by telephone call with the nephrologists, the dialysis unit’s staff, and the patients.

Our study has strengths. More importantly: an elevated proportion of patients has the diagnosis of bone Al accumulation, independently of renal osteodystrophy type, and this diagnosis was an independent predictor for MACE. Finally, we are proposing the new term “Al bone accumulation”, instead of “Al intoxication”, to refer the identification of Al in bone associated with non-severe symptoms (or subclinical manifestations) and outcomes.

## Conclusions

In this prospective cohort, an elevated proportion of patients have the diagnosis of bone Al accumulation, which was associated with a greater prevalence of bone pain, tendon rupture, and pruritus. Bone Al accumulation was associated with minor perturbations in bone dynamic parameters; no differences were noted in static bone parameters, the proportion of renal osteodystrophy types, and bone fractures. At follow-up, actual/prior diagnosis of bone Al accumulation and diabetes *mellitus* were independent predictors for major adverse cardiovascular events (MACE).

These findings suggest that bone Al accumulation may be considered a frequent and modifiable diagnosis in patients with CKD in the current Era, independently of overt Al toxicity. Efforts should be emphasized to improve water quality used for hemodialysis, as well quality of intravenous drugs and general measures to avoid Al exposition. More research is needed to understand the impact of consuming Al-containing food and water in patients with CKD, as well effects of early intervention for serum Al levels in patients with CKD.

## Supporting information

S1 Data (SAV)
